# Neural Basis of Solving Problems with Insight

**DOI:** 10.1371/journal.pbio.0020111

**Published:** 2004-04-13

If you're one of those insufferable people who can finish the Saturday *New York Times* crossword puzzle, you probably have a gift for insight. The puzzles always have an underlying hint to solving them, but on Saturdays that clue is insanely obtuse. If you had all day, you could try a zillion different combinations and eventually figure it out. But with insight, you'd experience the usual clueless confusion, until—voilà—the fog clears and you get the clue, which suddenly seems obvious. The sudden flash of insight that precedes such “Aha!” moments is characteristic of many types of cognitive processes besides problem-solving, including memory retrieval, language comprehension, and various forms of creativity. Although different problem-solving strategies share many common attributes, insight-derived solutions appear to be unique in several ways. In this issue, researchers from Northwestern and Drexel Universities report on studies revealing a unique neural signature of such insight solutions.[Fig pbio-0020111-g001]

**Figure pbio-0020111-g001:**
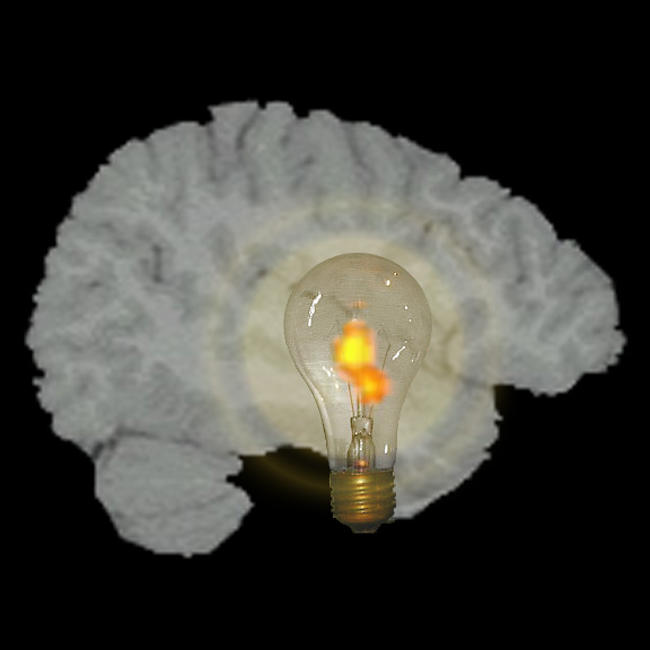
Insight lights up the brain

Mark Jung-Beeman, John Kounios, Ed Bowden, and their colleagues recount the storied origin of the term *Eureka!*, which Archimedes reportedly shouted upon realizing that water displacement could be used to compute density. Illustrating the strong emotional response elicited by such a sudden insight, Archimedes is said to have run home from the baths in euphoric glee—without his clothes.

Among other characteristics that typically distinguish insight from “noninsight” solutions, people feel stuck before insight strikes; they can't explain how they solved the problem and might say they were not even thinking about it; the solution appears suddenly and is immediately seen as correct. But are the neural processes involved in arriving at a solution through insight actually distinct from those related to more mundane problem-solving?

Recent findings suggest that people think about solutions, at an unconscious level, prior to solving insight problems, and that the right cerebral hemisphere (RH) appears to be preferentially involved. Jung-Beeman et al. predicted that a particular region of the RH, called the anterior superior temporal gyrus (aSTG), is likely involved in insight because it seems critical for tasks that require recognizing broad associative semantic relationships—exactly the type of process that could facilitate reinterpretation of problems and lead to insight.

To test this hypothesis, Jung-Beeman et al. mapped both the location and electrical signature of neural activity using functional magnetic resonance imaging (fMRI) and the electroencephalogram (EEG). In the first experiment, thirteen people were given three words (*pine*, *crab*, *sauce*) and asked to think of one word that would form a compound word or phrase for each of the words (can you figure it out?). Neural activity was mapped with fMRI while the participants were given 124 similar word problems—which can be solved quickly with or without insight, and evoke a distinct Aha! moment about half the time they're solved. Subjects pressed a button to indicate whether they had solved the problem using insight, which they had been told leads to an Aha! experience characterized by suddenness and obviousness.

While several cortical regions showed about the same heightened activity for both insight and noninsight-derived solutions, only the aSTG in the RH showed a robust insight effect. Given that neural activity in this area also increased when subjects first encountered the problem (perhaps reflecting unconscious processing), the authors conclude that the increase does not simply reflect the emotional jolt associated with insight.

In a second experiment, 19 new participants engaged in the same type of problem-solving tasks as the first group while their brain waves were measured with an EEG. The researchers then analyzed the EEG recordings to look for differences between insight and noninsight solutions in brain wave activity. The researchers found that 0.3 seconds before the subjects indicated solutions achieved through insight, there was a burst of neural activity of one particular type: high-frequency (gamma band) activity that is often thought to reflect complex cognitive processing. This activity was also mapped to the aSTG of the RH, providing compelling convergence across experiments and methods.

Problem-solving involves a complex cortical network to encode, retrieve, and evaluate information, but these results show that solving verbal problems with insight requires *at least* one additional component. Further, the fact that the effect occurred in RH aSTG suggests what that process may be: integration of distantly related information. Distinct neural processes, the authors conclude, underlie the sudden flash of insight that allows people to “see connections that previously eluded them.”

